# Supplementary material for: Metabolic Risk Factors and Survival in Patients with Glioblastoma
Source: Cancers (Basel). 2024 Oct 30;16(21):3666. doi: 10.3390/cancers16213666 (PMC11545693; doi:10.3390/cancers16213666)
Supplement: Supplementary file 1 [file cancers-16-03666-s001.zip › cancers-3218910-supplementary.pdf]

Supplementary Materials: Metabolic Risk Factors and Survival in Patients with Glioblastoma

John Paul Aboubechara and Orwa Aboud

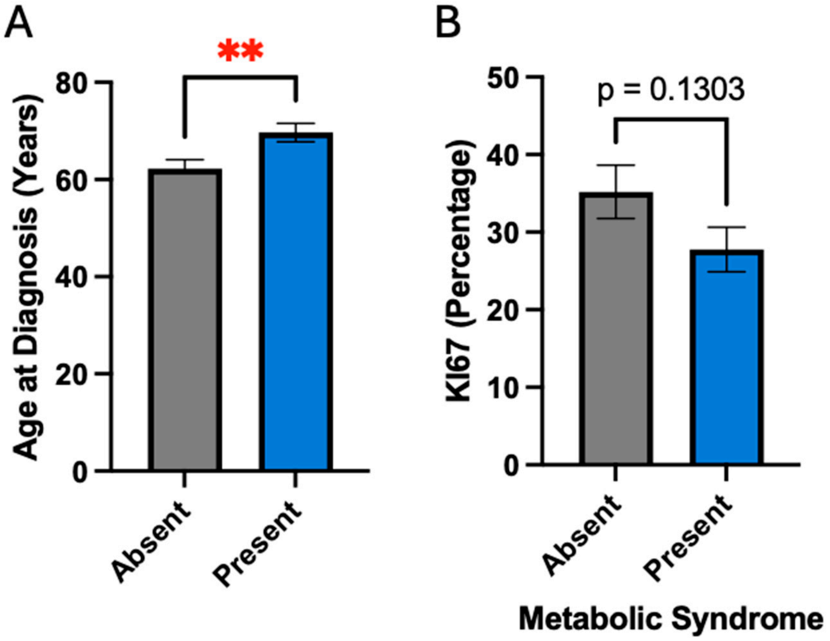

Figure S1. A) Patients with the metabolic syndrome were significantly older than patients without the metabolic syndrome. B) Patients with the metabolic syndrome demonstrated a trend towards higher KI67 index than those without the metabolic syndrome. ** signifies p-value ≤ 0.01
